# Challenges and Perspectives in Nutritional Counselling and Nursing: A Narrative Review

**DOI:** 10.3390/jcm8091489

**Published:** 2019-09-18

**Authors:** Maria F. Vasiloglou, Jane Fletcher, Kalliopi-Anna Poulia

**Affiliations:** 1 Diabetes Technology Research Group, ARTORG Center for Biomedical Engineering Research, University of Bern, Murtenstrasse 50, 3008 Bern, Switzerland; maria.vasiloglou@artorg.unibe.ch; 2 Nutrition Nurses, Queen Elizabeth Hospital Birmingham, University Hospitals Birmingham NHS Foundation Trust, Mindelsohn Way, Edgbaston, Birmingham B15 2WG, UK; jane.fletcher@uhb.nhs.uk; 3 Department of Nutrition and Dietetics, Laiko General Hospital of Athens, 11527 Athens, Greece

**Keywords:** nutritional counselling, nursing, interventions, e-counselling

## Abstract

Nutritional counselling has been recognised as the first line approach in the management of numerous chronic diseases. Though usually carried out by dietitians, nutritional counselling may be used by nurses, or other healthcare professionals to improve nutritional status and meet healthcare goals. Healthcare professionals require training and education to facilitate a patient centred approach to effective counselling. Advances in digital technology have the potential to improve access to nutritional counselling for some patients such as those in primary care. However, caution is required to ensure that valuable interpersonal relationships are not lost, as these form the cornerstone of effective nutritional counselling. The aim of this narrative review is to explore aspects of effective nutritional counselling, including advances in e-counselling and areas where nursing input in nutritional counselling might enhance overall nutritional care.

## 1. Introduction

Nutrition related chronic diseases—i.e., cancer, diabetes mellitus, chronic kidney disease, inflammatory bowel disease and so forth—and health problems that have significant implications on nutritional status, mainly by affecting digestion and absorption of food, place a significant burden on the overall health of a population and health care systems. As dietary modification can have a significant impact on biomarkers of non-communicable diseases and on symptoms of different clinical conditions, nutritional counselling has been recognised as the first line approach for their management [1]. Furthermore nutrition counselling is recommended as a key intervention in the management of malnutrition in older adults [2,3], and has been proven to be effective in chronic kidney disease, cancer and other clinical conditions [4,5,6].

Nutrition counselling is a two-way interaction through which a patient and the member of the medical team interpret the results of a nutritional assessment, identify patient’s nutritional problems, needs and goals, discuss ways to meet these goals, and agree on future steps and the frequency of monitoring. It aims to help patients understand important information about the impact of nutrition on their health status and focuses on practical measures to cover nutritional needs. Moreover, it strengthens the importance of behavioural change [7,8,9,10]. However, nutrition counselling may present a time burden to patients, with high drop-out rates being a key challenge likely to affect outcomes [1], and face to face counselling having a significant impact on the use of resources in terms of clinic space and facilities. The development of new technology in e-counselling seeks to address these issues and expand access for patients.

Most of the time, nutrition counsellors are nutritionists or dietitians. However, other healthcare professionals such as nurses, community health workers or volunteers [3,7,11] play an essential role in nutritional counselling. The role of the nurse is recognised in nutrition screening but there is little evidence regarding the impact or effectiveness of the nurses’ role in nutrition counselling. However, nurses form a core part of the team providing direct care to patients and as such are in a position to make positive behaviour changes [12], improving the effectiveness of nutritional interventions. 

To meet the needs of an ever-changing healthcare environment, there is a need to explore the benefits of new technology in providing nutritional counselling to a wider audience. Although dietitians are key professionals in the nutritional counselling of patients, expanding the roles of other healthcare professionals may improve the impact and effectiveness of both counselling and interventions. 

### Aims

In this narrative review we aim to explore aspects of effective nutritional counselling including advances in technology and the role of the nurse in enhancing nutritional care.

## 2. Methodology

An electronic search was implemented in PubMed, Google Scholar Medline and Cinahl databases. Search terms included: ‘Nutritional counselling’, ‘nurs* AND nutrition counselling’ ‘dietary counselling’, ‘patient education’, ‘nutrition education’, ‘nutrition AND nurse AND specialist’ ‘nursing’, ‘e-counselling’, ‘mHealth’, ‘eHealth’, ‘nutrition nurse specialist’, ‘patient-centred approach’, ‘nutritional care’, ‘remote nutrition counselling’. Publications were only included in the analysis if they were written in English and were related to humans. Articles were searched from January 2000 till May 2019. Non-peer reviewed literature, letters to the editor and studies performed on animals were excluded. From the articles retrieved in the first search round, additional references were identified by a manual search among the cited references. Moreover, additional references including characteristic and cornerstone references on nutrition counselling were added in introductory parts. As the review is narrative and not systematic, the references were selected according to the relevance to the subject of the manuscript.

## 3. Basic Characteristics of Effective Nutritional Counselling

As nutrition counselling can enhance health and nutrition outcomes, ideally it should be performed in a way that ensures patients’ privacy and that the counsellor and the patient are feeling comfortable. This may be challenging in the hospital setting and measures should be taken towards this direction. Moreover, counsellors should be trained and use appropriate materials to enhance understanding and documentation, such as illustrations, food models, take-home brochures, data collection forms and referral forms.

Moreover, respecting the ethical aspects of nutritional counselling improves its outcomes. The main ethical values that should be followed are described in Table 1:

### Patient-Centered Approach in Nutrition Counselling

The implementation of changes on nutrition behaviour is a complex procedure, as it combines elements of psychology, physiological needs, socioeconomic status and the level of the counsellor’s ability to firstly identify the needs of the patients, and then to work co-operatively with them [3]. The patient-centred approach is generally accepted as efficient in managing nutritional counselling. It has been developed based on theoretical models developed in the late 40’s by Rogers [13]. According to this theoretical model the patient has to identify the diet related problems and the nutritional counsellor will guide him towards the possible solutions.

The nutritionist/dietitian is the expert in the medical nutrition team, able to translate and combine the most current scientific information on food and health, food composition, psychological and physiological factors that could have an impact on dietary choices, and the relationship of those with health and diseases. He/she is the nutrition communicator, a skilled listener and a translator of emotions and abstract ideas on specific actions and steps towards dietary modification needed to enhance nutritional status, based on the individuals’ life and biomedical requirements [14,15,16]. Gaining a better understanding of patients’ preferences, attitudes and beliefs regarding their state of health and nutrition would allow interventions to be more focused, appropriate, sustainable and therefore, more effective [17]. The clinician–patient relationship has been associated with better patient attendance and adherence and greater patient satisfaction with care and treatment [18]. Moreover, patients’ recall understanding, treatment adherence, and psychological wellbeing have been associated with effective doctor–patient communication. Therefore, an effective counselor refers not only to the expert nutrition knowledge but also to the privilege of having good communication skills. In this way, the development of a therapeutic relationship, characterized by mutual respect with the clients, will be easier to manage. However, this mode of practice requires organizational support and time, which are not always available to the dietitian in clinical practice. These dimensions may also be important in nutrition interventions, and future research should focus on the efficacy of nutritional counselling with these limitations in mind [19].

Under this spectrum, the main aim, and yet a great challenge, is to ensure that the patient has a full understanding of the relevant information of the nutritional problem and can work towards the development of specific skills to deal with the health challenges and their personal goals [20]. In order to have a successful outcome in patients receiving nutritional counselling, it is important to assess not only the patients’ food choices and behavior, but also their access to health, environmental and social support systems. Therefore, the challenge is to be able to achieve essential and meaningful clinical outcomes, enhance the quality of life of the patient and, at the same time, encourage positive attitudes towards behavioral changes [21].

## 4. Challenges in Nutritional Counselling 

Health professionals should always have in mind that patients are members of the community at large. At the community level, they receive multiple messages from different sources regarding nutritional behaviors that could enhance their wellbeing, their quality of life and improve health outcomes. Although health outcomes can be estimated by objective measurements, in terms of biochemical and physiological responses to interventions, the main issue is to identify what patients consider important as endpoints of quality of life and wellbeing [21]. Under the spectrum of the patient-centered approach, the bridging of biomedical imperatives and individual perspectives on health, quality of life and wellbeing is of paramount importance. 

According to a recent systematic review by Mitchell et al. [1] of 26 randomized controlled studies (RCTs), representing 5500 adults, dietetic consultation in primary care appeared to be effective for the improvement of diet quality, the glycaemic control, and body weight control. One major limitation in analyzing RCTs that deals with nutrition consultations and dietary interventions though, is the fact that dietary assessment is subjected to errors and the available dietary assessment tools may not be too specific to identify qualitative improvements to the diet. Moreover, as nutrition is a factor influencing patients’ life to a great extent, and at the same time is being influenced by personal, economic, psychological and emotional reasons, dietary interventions are usually characterized by high dropout rates and limited compliance. Therefore, measures to enhance adherence to dietetic interventions should always be considered [1].

Moving to more clinical settings, dietary interventions have been studied in environments that compromised health has a negative impact on nutritional status and raised the risk of malnutrition. In clinical cases health professionals providing nutrition consultations should always follow evidence-based medicine and established guidelines. As nutrition can be used as a complementary treatment that could be even harmful in some cases, i.e., in cancer patients, nutrition counselling should always be provided by specialized and qualified nutrition and dietetics professionals, to ensure the validity of the provided information. Lack of knowledge and misconceptions should be pointed out and scientific evidence should be provided. All non-justified and not scientific-based dietary theories should be addressed, under the spectrum of the individual perception of wellbeing [22,23].

In order to enhance compliance and adherence to nutritional interventions, other factors that could have an impact on the effectiveness of the intervention must be considered. The duration and the frequency of the consultations, the aims set on each consultation, the feedback that was given to the patients about their improvement or the achievement of the set goals can have an impact on the adherence to the nutritional intervention. More focused, clearly defined and measurable objectives increase effectiveness, whereas the use of vague objectives may limit the effectiveness of nutrition education due to the fact that it creates confusion and does not provide answers to the questions of the patients. Therefore, the design of the interventions should be clear and continued training for the trainers, with an emphasis on targeted interventions, should be available [24,25].

## 5. Nursing and Nutritional Care

Where studies have shown that patients benefit from receiving counselling and an individualised nutrition plan from a registered dietitian [26,27], the nurses’ role is essential in reinforcing and facilitating nutritional plans and interventions in hospitalised patients. Observations suggest that nutritional outcomes may be enhanced with collaborative input from both dietitians and nurses [28]. As the daily providers of care, ward-based nurses are responsible for the direct delivery of nutritional interventions [29]. Additionally, nurses have a responsibility to pay special attention to promoting wellbeing, with nutrition being one of the fundamentals of care [30].

### 5.1. Ward-Based Nurses

Perry et al. [31] report a systematic review identifying the range of nursing interventions implemented to improve nutritional outcomes in patients who have suffered a stroke. The review included 27 papers from 26 studies with five RCT’s and five clinical trials. Due to the heterogeneity and poor quality of reported data, few interventions showed statistical significance. However, this review is important in highlighting the diversity of nursing activities in nutritional care. The Alliance to Advance Patient Nutrition [32] stresses the importance of multidisciplinary involvement in nutrition, with a focus on all stakeholders valuing nutritional care interventions. Important points for nursing intervention include nutrition screening, implementing early nutritional measures in those at risk and developing strategies to ensure patient compliance. Where dietitians carry out comprehensive assessment and patient education, the nurse’s role is to reinforce this information and answer patient questions [32]. In this way, collaborative working between disciplines is essential. 

Nevertheless, one of the key challenges in practice is the level of importance that nurses place on nutritional care. A study of the attitudes of 106 nurses found respondents felt strongly that it was the role of the nurse to carry out nutrition counselling [33]. Key nursing nutritional interventions were significantly positively associated with the overall nutrition knowledge score: Patient counselling (*r* = 0.23, *p* = 0.02) and nutrition screening/assessment (*r* = 0.23, *p* = 0.02). This finding indicated that the higher the overall nutrition knowledge score among nurses, the higher these tasks were ranked [4]. However, the same study found that nurses ranked tasks associated with nutrition lower than other nursing tasks, such as giving medication and carrying out wound dressings. A small (*n* = 22) focus group study of staff working in an acute medical ward found that nurses struggled to put nutritional priorities in to practice, with competing activities being a particular issue at mealtimes [34]. A nursing survey carried out in Canadian hospitals (*n* = 346) reported nurses’ perceptions as one of the reasons for insufficient nutrition [35]. The study found that 17% of the respondents thought that a lack of assistance at mealtimes was a major contributor, and 14% thought that assisting with nutrition was time consuming [35]. 

Though nursing interventions such as screening and assistance with eating and drinking are crucial, there appears to be little recorded evidence of nurses carrying out nutritional counselling at the ward level in internal medicine. Studies suggest successful nurse involvement in nutritional counselling in other settings including cancer care [36,37], primary care [38] and community care [39,40]. This then suggests that nurses working in internal medicine may be missing a valuable opportunity to improve patient care and compliance with nutritional interventions. 

### 5.2. Nutrition Nurse Specialist

Specialist nurses have an important part to play in empowering ward-based nurses to deliver evidence-based care within their speciality, bridging the gap between research and bedside practice [41]. The nutrition nurse specialist (NNS) role may be known by numerous titles including ‘nutrition support nurse’, ‘nutrition nurse practitioner/advanced practitioner’ and ‘nutrition support consultant nurse’ [42]. Regardless of title, NNSs generally form a core part of hospital nutrition support teams, caring for patients with complex nutritional requirements. Though it is difficult to ascertain the exact value that individual members of the multidisciplinary teams bring to nutritional support, input from NNSs have been shown to reduce catheter related sepsis and other complications of parenteral nutrition [43]. 

However, the NNS role reaches far beyond this. The NNS may coordinate patient education on self-management of enteral or parenteral nutrition and provide training and educational programmes for other healthcare professionals [42]. Yordy et al. [44] report outcomes of a NNS-led quality improvement project. The project included a program of nutritional education for ward nurses with an audit of practice before and after education. In observations of mealtime interactions (*n* = 100), patient interruptions at mealtimes were shown to have reduced by 59% following the education program. Nursing documentation of nutritional risk factors and screening showed some improvement after education, although no statistical significance was described. The authors suggest that following targeted education, the nursing culture had changed to recognize nutrition as an essential part of the patients’ treatment [35]. 

Some NNSs may be involved at a strategic and organisational level with the provision of nutritional services within their institution. Depending on the nature and requirements of the healthcare setting, NNSs may expand their skills to include technical procedures such as central venous catheter insertion [45] or gastrostomy insertion. Their scope is expanding further to include independent non-medical prescribing [46]. However, there is a lack of evidence regarding the function of NNSs in nutritional counselling. Given the nature of the role, it is reasonable to expect that NNSs play an important part in counselling patients with complex nutritional needs and particularly those that require artificial nutrition support. For these reasons NNSs must develop effective counselling skills. Fundamentally, the overall purpose of the NNS should be an improvement in quality of nutritional care, and nutritional counselling must be a core activity in this.

Further research is required to measure the improvements that the enhanced skills of the NNS brings to nutritional care. Nevertheless, NNSs are key participants in the wider nutrition support team and have an important function in supporting and educating ward-based nurses to deliver excellent care and reinforcing multidisciplinary nutritional treatment goals.

## 6. Nutritional Counselling with the Use of Modern Technology

Nutritional counselling is usually a time- and resource-demanding procedure that requires commitment by the patient [47]. The dropout rate of up to 35% of patients enrolled in dietetic consultation interventions shows the challenge of adhering to numerous counselling sessions [1]. Modern technology, including electronic health (eHealth) and mobile health (mHealth) may help keep patients engaged in their goals [48]. According to the European Commission’s definition, eHealth is the “digital health and care which refers to tools and services that use information and communication technologies (ICTs) to improve prevention, diagnosis, treatment, monitoring and management of health and lifestyle”. mHealth is a subcategory of eHealth, used mainly for describing healthcare management conducted by smartphones [49].

Electronic or e-counselling, i.e., remote counselling, can reduce barriers related to patient disengagement, geographical distance, time constraints [48], socioeconomic status [50], and also low need or desire for in-person contact [47]. In addition, the integration of eHealth and mHealth technologies could render the flexibility of dietetic services administered to patients [51,52]. Smartphone applications (apps) have been used of late to improve nutrition knowledge and contribute to behavioural change (beyond weight loss) [53], while presenting positive effects on measured nutritional outcomes in chronic diseases [47]. Artificial Intelligence (AI)-based smartphone apps can provide accurate and almost real-time dietary assessments [54,55,56,57,58,59]. However, even though there is an abundance of nutrition and diet apps, the majority of them focus on diet monitoring and nutrient content estimation. There is only a limited number of studies investigating the long-term impacts of apps focusing on nutritional e-counselling [52].

E-counselling through web-based apps has also already produced some positive effects, in comparison to in-person treatment for both delivery and dissemination [60]. Weight loss of approximately 4–6 kg can be reliably achieved with web programs that involve some form of weekly human e-counselling or constant feedback from behavioural lifestyle counsellors via email, group chat, etc. [61]. The results of this study also suggest that using a hybrid approach (in-person and online) may be an effective way to reach larger and more diverse populations [61]. Weight loss targeted web applications, where participants also received e-counselling via weekly e-mails, resulted in weight loss of 4.4 kg after one year of intervention in people at risk of type 2 diabetes [62]. Providing automated, computer-tailored feedback by a weight loss targeted web application for three months was as effective as human counselling via e-mail [63]. Clinically significant weight loss could be achieved through a combination of tailored goals and the use of a mobile app, whereas people interested in lower-intensity weight loss approaches could consider stand-alone digital health treatments [64]. Furthermore, Haas et al. [50] concluded that an app which complemented the dietitians’ professional skills, can provide effective support towards behavioural change and sustainable weight reduction in overweight and obese individuals. Moreover, traditional consultations delivered by dietitians were compared against remote ones delivered by eHealth technologies, using theoretical cost in weight management as the outcome measure [52]. The eHealth approach needed an initial higher investment but was less costly in the long-term than in-person counselling [52].

In a qualitative study, healthcare professionals stated that it was challenging to establish and maintain an empathetic relationship with their clients (which is one of the most crucial factors for coaching) when conducting eHealth counselling [65]. Hence, e-counselling should include specific attributes that ensure it simulates—as closely as possible—a face-to face consultation [50], tailored to the individual patient [65].

Finally, including innovative technologies into dietetic practice could assist nutritional counselling by not only enhancing the efficiency and quality of nutrition care but also increasing adherence to self-monitoring of patient-centred goals [66]. Currently there is no evidence on the most effective use of apps in a clinical setting. Thus, it is suggested that the apps are used alongside individualized dietetic support, while performance for long-term weight management is being assessed [67]. End-users (patients) should be involved in the design process concerning health advice since it increases app efficacy and usability [68]. Future research, preferably in the form of randomized controlled trials, should investigate the clinical efficacy, feasibility and cost-effectiveness of e-counselling and eHealth technologies on dietetic practice.

## 7. Conclusions

Nutritional counselling has been recognised as the first line approach in the management of numerous chronic diseases. A patient-centred approach has been identified as the best way of providing nutritional counselling. Ideally, it is carried out by dietitians and nutritionists, but other members of the medical nutrition team such as nurses and other healthcare professionals can also play an important role. Nutrition nurse specialists have a particularly key part to play in both the carrying out of nutritional counselling as well as encouraging other nurses to participate in nutritional counselling. However, research is required to fully understand the benefits that the NNS brings to nutritional counselling and care. 

It is important to stress that effective counselling needs training and education, and at the same time the use of eHealth technology has the potential to improve access for some patients. However, caution is required to ensure that valuable interpersonal relationships are not lost, as these form the cornerstone of effective nutritional counselling. 

## Figures and Tables

**Table 1:** Ethical values that underpin effective counselling [4].

Ethical Values	Rationale
The provision of accurate information	Patients should develop a relation of trust with the counsellor, based on the fact that the words and actions are true and reliable.
Confidentiality	All information shared should be kept confidentially, except as needed for the nutritional treatment and recovery.
Respect of patients’ autonomy	Patients, as long as they are mentally stable, keep the right to decide for themselves, without coercion.
Do no harm	Nutritional interventions in the hospital setting should always be based on evidence-based medicine. Any intervention that could harm or exploit patients emotionally, financially or medically should always be avoided.
Be fair	All patients should receive the same level of attention, according to their needs, without discrimination. Patient’s rights dignity and differences should be respected.

## Abbreviations

The following abbreviations are used in this manuscript:

NNS	Nutrition nurse specialist
eHealth	Electronic health
mHealth	Mobile health
e-counselling	Electronic counselling
ICTs	Information and communication technologies
